# Reflective practitioners: The importance of critical thought for change agents

**DOI:** 10.4102/jamba.v7i1.128

**Published:** 2015-02-27

**Authors:** Terry Gibson

**Affiliations:** 1 Global Network for Disaster Reduction, London, United Kingdom

## Abstract

Investment of resources in international development continues to grow, but evidence suggests that progress is patchy and that parameters such as losses resulting from disasters of all scales continue to grow. It is suggested that critical thought and reflection, as an adjunct to action, is vital for both individuals and organisations concerned for social development. It is further argued that disruption is often required in order to dynamically pursue transformation of structures and institutions in order to secure progress towards sustainable livelihoods.

## Introduction

I am honoured to be giving the Pat Reid memorial lecture at the kind invitation of the African Centre for Disaster Studies (ACDS), a great team who have collaborated with us for over 5 years now at the Global Network for Disaster Reduction (GNDR)^1^ – in fact, over the whole of the network's active life. A little more of what the network does later; for now, just to say it links together civil society organisations along with academics and others all over the world to advance the cause of disaster risk reduction (DRR), reducing people's vulnerability to disasters.

I was working with a group of our members in Amman, Jordan a couple of weeks ago and in discussion with our adviser, Ben Wisner, who was also present, I mentioned this lecture. His eyes lit up at the mention of Pat's name. ‘A great and inspiring person’, he said. ‘Very practical, and through dogged persistence instrumental in getting DRR enshrined in law in South Africa.’

Praise indeed coming from Ben, who is in fact one of the founding fathers of DRR. It was also, I have to confess, my first introduction to Pat. I immediately offered requests for guidance and enlightenment about Pat to the Great Google God, and thankfully they were forthcoming.

Therefore I understand that in giving this lecture I am stepping respectfully and humbly into the footsteps of greatness – of an activist who made a difference. My subtitle is ‘the importance of critical thought for change agents’ and this lecture is about that theme – *activists* who make a difference.

I am embarking on this theme in the presence of many who in one way or another are concerned about change, about development. As such we are part of a huge history which, at least since the foundation of the modern developmental organisations and banks at Bretton Woods post-war, has expended a huge amount of effort and invested huge and growing sums to achieve social change and progress. Over those 50+ years the passionate work of many at every scale from local to global has clearly made a huge difference. One of these was Pat.

## The challenge for change agents

But, and there is a huge but, the fact is that at global, national and local level many of the trends are negative rather than positive.

Globally, the figures for overall aid expenditures have grown and grown. In 2010 alone, total development expenditure was $509 billion.^2^ However, at the same time erosion of livelihoods is increasing rather than decreasing,^3^ the figure for global losses from disasters reaching $350 bn for natural disasters alone in 2010, and doubling every 20 years. This despite the fact that the UN 10-year programme for Disaster Reduction – the Hyogo Framework for Action^4^ – which concludes next year, has as its expected outcome ‘the substantial **reduction** of disaster losses, in lives and in the social, economic and environmental assets of communities and countries’.

GNDR's own research shows that it is the poor who are hit hardest, with its large-scale views from the Frontline social survey showing that only those who regard themselves as wealthiest think losses are decreasing.^5^ All other economic groups believe they are increasing. On this and other measures the international system and the nations it comprises seem powerless to achieve the step change required to break through the barriers.

If this is a challenge for GNDR as for other *global* change agents, then it is, too, for those at other scales. At regional and national level the role and the scale of civil society has grown dramatically, in part as it has filled the vacuum created by the erosion of social protection resulting from shifts in economic strategies. However, as I sat with several colleagues – energetic activists from civil society organisations in Nigeria and Cameroon – they bemoaned their inability to operate as ‘sustainable NGOs’, free to take steps to create change and progress. Why? Because they are dependent on donors who shackle them to project cycles, constraining their activities and boxing them in to 1-year, 2-year or 3-year cycles of action. They know that real, sustainable change takes much longer than that. This is a familiar story for civil society organisations ranging from small in-country civil society organisations (CSOs) to large international non-governmental organisations (INGOs). It militates against strategic long-term steps designed to break through the barriers.

If civil society is locked into cycles of action and is unable to think strategically and critically, perhaps it should turn to the calm, reflective world of academia? Making that suggestion in a UK academic setting would be guaranteed to trigger a dry, ironic chuckle. Why? Because many academics describe themselves as similarly shackled to funding and evaluation cycles. I am an external associate of a university research centre, and I suggested a piece of work which fitted exactly the goals of that centre. The response from the centre's head (pers. comm., 17 October 2011) was:

Unfortunately it falls, for us, at the wrong time in our government-imposed academic cycle. All staff have to focus on high-quality academic publication for the next 18–24 months, and we have to turn aside from pretty well any other work. So while this was just the sort of thing I set up [*the unit*] to be doing, unfortunately in practice we are unable to help.

If activists in both civil society institutions and academia are shackled through structural factors to an uncritical status quo, what about the local level? What are the opportunities here to break through the barriers?

GNDR works through civil society with communities in over 70 countries and finds tremendous energy and insight amongst them. However, there is also dependence and passivity, ingrained by the structures around them. Spending time with a local CSO in rural Ethiopia, people in the villages repeated the following saying: ‘We won't go hungry as long as there is a good grain harvest in Canada.’ Years of dependency on emergency response to famine had led communities to learn that sacks would generally appear, normally labelled ‘Canada’ – the source of much World Food Programme grain. They had become shackled to a system, unable to see a way to break through the barriers.

## Failing to break through the barriers

So here is the problem. Many recognise the need to break through the barriers and achieve substantive change and progress. International institutions such as the UN body responsible for disaster risk reduction – The United Nations Office for Disaster Risk Reduction (UNISDR) – recognise the need for change. Its 168 signatory countries share that vision. Civil society organisations bring their own perspective for change in policies, resourcing and action. Academia provides thoughtful reflective analysis of systems and structures. People at local level often have clear and insightful views on the options for progress. However, in many cases all these actors are limited to a dogged activism – activism with a huge price tag attached, but which seems unable to achieve step change, breaking through the barriers. Change agents at all levels – international organisations and frameworks, government, civil society, academia, local communities – find themselves confounded by the ‘Red Queen hypothesis’. Like the Red Queen in *Alice in Wonderland* they are running and running and standing still. Resources, energy and activism are invested in huge measure at all levels, and yet we run and run and stand still. We fail to break through the barriers.

At this point I have set myself up to fail by presenting a huge and intractable problem with which many of you may empathise within your own professional practice. Many of you may recognise the ‘Red Queen hypothesis’ as ringing true to your experience. Many may see that institutions at global, national and local level face this challenge. So where is the quick fix? How can we unlock the energy of change agents at all levels and create what is sometimes called ‘transformation’ – fundamental change in options for progress? What would free up CSOs and academics to identify and act out options for substantive change? How could governments and international frameworks create real tipping points which broke through the barriers and changed the trajectory? That is quite a big question.

I will come clean and say I do not have *the* answer. But I do have *an* answer. It is an answer which is discovered in diverse locations, applying to diverse areas of practice, offered by diverse thinkers into diverse disciplines. What interests me is that whilst the answer is therefore dressed up in different guises, using different words and methodologies, at its root it is – in all these shape-shifting identities – the same answer. It can be applied to individuals, to groups, to civil society professionals, to academics, to those in government and to those in international institutions. I have come across this answer in many different arenas. Originally a biologist and a philosopher of science, I worked for many years in business and in the media. More recently I have worked in the field of international development, moved on to study and research, and most recently worked closely with civil society in GNDR. In science, philosophy, business, media, academia and international development I have come across the same answer in different disguises.

## Options for change

The answer comes from Habermas (1987), Polanyi (1958) and others in the world of philosophy, Lewin (1946), Dewey (in Kolb 1984) and Lave and Wenger (1991) in the world of informal education, from Kolb (1984), Checkland and Scholes (1999) and Revans (1982) in the world of industry, from Nonaka and Takeuchi (1995), Senge (1990) and Christensen and Raynor (2013) in the world of innovation, from Argyris and Schon (1974) in the world of professional practice and from Ostrom (1990), Long (2002), Wals (2007), Gaventa (2005), Chambers (1983) and finally Freire (1996) in the world of international development. My point in offering this verbal bibliography is simply that this is an idea which emerges from diverse worlds, wherever the role of learning in change is recognised.

Many names, diverse disciplines, very similar messages about how – whatever the challenge – to break through the barriers which prevent change, enabling progress and transformation, whether at global, national or local level. The answer, suggested by the title of this article calling for change agents to be reflective practitioners, is what I will call critical thinking.

All the above thinkers, in different ways, characterise the power of individuals and groups moving beyond activism to action and reflection, thinking critically about their work and identifying new possibilities for action. Let me bring this to life with a couple of examples, one global and one local.

## Critical thinking and global debt

We started with intractable curves, and we will take as an example of learning and change at global level another intractable curve from the 1990s – the levels of debt faced by many countries in the developing world. You may remember the growing impact of years of high interest rates in the 1980s and 1990s. Many newly independent nations had welcomed the cash on easy terms which came in many cases from the Organization of the Petroleum Exporting Countries (OPEC) nations awash with cash after the fuel price hikes of that time. Soaring interest rates meant these loans quickly became a ball and chain, dragging countries back. The wealthy nations offered them terms, but they were not easy. ‘Structural adjustment programmes’ held an economic gun to the countries’ heads, demanding large-scale cutbacks in education, social protection and state industry in return for debt relief. I was in Zambia as health charges were imposed in rural healthcare dispensaries, effectively barring the poor from even the most basic healthcare. Regardless of politics it was widely acknowledged that the debt crisis and the programmes that followed were causing large-scale human suffering. The global big players – the International Monetary Fund (IMF), World Bank, G7 – seemed totally resistant to change.

In 1990, a lecturer at UK's Keele University, Martin Dent, hatched a scheme to mobilise students to address this, based on the Biblical principle of Jubilee, an Old Testament form of debt remission. With Bill Peters and Isabel Carter they recognised that their very simple message could capture public imagination to address this.

Over several years they enlisted the support of large INGOs who had previously seen no way they could achieve transformational change, and by 1998 they were able to take 70 000 peaceful protesters to Birmingham to form a human chain (the symbol of Jubilee 2000) round the G7 meeting there. Running scared, the organisers changed the venue of the meeting to avoid publicity but the event attracted so much media attention that Prime Minister Blair had to fly back to Birmingham from the new venue to meet with protesters and organisers.

By 2000 the campaign was international, supported by millions of campaigners along with high profile politicians and celebrities. Ultimately it led to the remission of debt, either partially or totally, in several countries (although the promised remission was more partial in practice than was promised). The campaign had achieved unimaginable political influence and change. Contained in that story are several key ideas for change agents – ideas shared by all the thinkers I mentioned above.^6^

Firstly, the role of critical thought. Critical thought steps outside the system, draws on new ideas, and identifies a new way of achieving change. Freire (1996) showed how people in poor Brazilian communities could become critical of their seemingly intractable situation and work out ways to break through the barriers. Martin Dent drew on a very different world – that of Old Testament law – to inspire his idea. He applied it to an intractable system, and that leads to the second idea.

Critical thinkers need to look at the whole system to identify options for change. Senge (1990) argues that in organisational change ‘systems thinking’ is the key. Checkland and Scholes (1999) add the word ‘soft’, showing how in the real world we need to understand complicated systems with power dynamics, emotions, and many different drivers to work out what the entry points are for change.

Dent, Peters and Carter were change agents who took their role seriously. Schon (1983) argues that true practitioners in any discipline – ‘reflective practitioners’ – have a professional duty to be continually questioning and critical. Too often, he says, in our technocratic world people simply operate within established processes and systems rather than taking their duty to reflect, think critically and question seriously.

Thinking critically about the system leads to identifying options for change which leads, according to Christensen and Raynor (2013), to ‘disruption’. By this they do not mean bloody revolution. They are talking about innovation and growth. What they are talking about is the conditions for step change, and the necessity for step change to allow innovation and progress.

Interestingly they assert that whilst disruption is essential for innovation and progress (think of great disrupters like Amazon, Google or Facebook who have changed the game) an organisation cannot be its own disrupter. This is pretty fundamental. A professor colleague says when people at his university he has thought of as activists go off to the World Bank to ‘change the system from within’ he does not believe them. The system is stronger than they are. An easy jibe, meeting them several years later in a Washington apartment at a canapé party, is to ask if they are still changing the system from within? They are not. The information technology giants I mentioned above all originated as tiny start-ups. Microsoft, which started similarly, struggles to hang onto their coat-tails even with massively expensive acquisitions.

More broadly, at every level the very qualities that bind communities and organisations together, their shared values, knowledge and culture, are what make change so difficult. The activist philosopher Habermas (1987) refers to ‘the reservoir of taken for granteds’ – the body of ideas which are accepted and often unspoken. Because this shared view of the world is implicit – taken as read – it is hard to influence it and where change is needed disruption of the established way of seeing things is almost always necessary.

This leads to a final point. Critical thinking is about more than action and disruption; it depends on the duo of action and reflection. Kolb (1984) enshrined this idea in his ‘learning cycle’ model. At its simplest it is learning by doing, with the key step of questioning what you have done and using that questioning to do it better, differently or even not at all (Kolb 1984).

For Jubilee 2000 as an organisation, grappling with critical reflection on their action was a step too far. This great action climaxed in 2000 with events such as the G7 summit in Cologne. The campaign had become global. As campaigners marched in Germany, I was on the southern borders of Zambia filming villagers taking part in Zambia's J2000 campaign. Then, the contracts for the organising team expired on 31 December 2000. However, 8 years later a celebration of the Birmingham human chain was staged to a modest audience by the ‘Jubilee Debt Campaign’ – a small and shrinking organisation struggling to find a niche. Learning by doing demands critical reflection on what has been done and what is learnt about what to do next. A strong argument in that case might be that the answer was to stop.

One final point about Jubilee 2000: I mentioned that Chris­tensen and Raynor argue that an organisation cannot be its own disrupter. This was true of the INGOs who rallied to the campaign. Research into Jubilee 2000 reveals the tensions between those INGOs and the campaign itself (Clark 2003). Was the messaging theoretically and politically correct? Were they comfortable with the strategy? Was the energy devoted to the campaign distracting supporters from supporting their own programmes? The tensions and the cracks grew, the uncomfortable alliance was only held together by the scale and importance of the campaign, and rather like a nuclear reactor, once the containing force of the campaign was removed in 2000 the consortium blew apart. This adds force to the idea that change agents need to be creative and create new spaces for change. Gaventa (2005) provides the ‘power cube’ framework for applying this politically at local level, showing how change often depends on rejecting ‘invited’ political spaces where the hosts have all the power, and creating new political spaces instead. To use another Biblical metaphor, you cannot put new wine in old wine-skins.

## Critical thinking by practitioners and academics

What about worlds closer to our own – the worlds of academia and civil society? You can see already that there is a big question here about systems. Civil society practitioners see themselves as activists, and look to academics to do the thinking. At least that is one view; another is that each mistrusts the other. We staged a workshop last year at a London university where scientists, technologists and civil society practitioners met and shared their case studies. Over 2 days there was a thawing as the civil society activists realised the scientists had really interesting ideas that could inform their work. The scientists, conversely, realised that their ‘big science’ needed to engage with the real world and the practical knowledge of the practitioners to have traction.

If academia is a system which might require disruption, then so is civil society. Its activists often ignore or even resent critical thought from academics about their actions. For example, in one recent online debate between a number of UK-based INGOs, who had seen an academic paper which was critical of their strategy, one wrote:

I'm a bit baffled by people who think that the word is so flawed that we need to reject it in favour of some better one – quenching the ‘buzzword’ energy, rather than trying to channel it appropriately while it's at its strongest. (J. Hafvenstein, pers. comm., 22 October 2012)

The ‘buzzword’ in question was ‘resilience’ – a widely used and misused word. Another contributor wrote: ‘Surely it is up to us to define the parameters of resilience work in practice (rather than theoretical and academic debates)’ (D. Hilier, pers. comm., 23 October 2012).

Schon (1983) argues that all practitioners have a duty to be open, reflective and critical, rather than getting their heads down and simply rolling with the buzzword energy (Schon 1983). Pulling up the drawbridge and rejecting theoretical and academic debates in favour of dogged activism contributes to throwing increasing resources at situations without ever achieving real change.

My final example of a reflective and critical approach playing out to achieve change at a very different level is in rural Ethiopia – where I had been told that people were often passively resigned.

## Critical thinking in a village community

Gale Warego is a village in the Wolaitta region of Ethiopia, about 5 hours’ drive from Addis Ababa. The villagers have an agrarian subsistence lifestyle. Apart from expecting sacks of grain when famine hits, the villagers had a clear list of requirements which they had shared with the local non-governmental organisation (NGO) – things like a dispensary, a school, a veterinary centre and agricultural inputs such as fertilisers. Not only had none of these been provided, but the local government never even visited the community spread along the valley and the hills to discover their needs as access was too difficult. As a result the villagers were relatively passive and fatalistic.

The NGO staff undertook a course in a learning method called ‘self-organised learning’. It has much in common with the approaches to critical thinking which I have mentioned, placing an emphasis on understanding the whole system and thinking critically about the options for change. The workers shared this method, in turn, with the villagers. As they discussed the problems they faced, employing this critical thinking process and looking at their whole situation, they realised that the real key to progress was a road. If there was a road through the ribbon community then access would be created and would open up all the other possibilities, starting by increasing the likelihood that the local government officers would come to the area.

The solution led to further challenges. How would the road be built? No one else would do it. They had to. They planned work teams so that everyone gave a day a week in separate groups to the project. Even with willing person-power a further problem remained. The road ran through mostly private land. They had to persuade the owners of the value of giving up parcels of land to the project. All these problems were solved and when I visited, the road construction was well underway. What is more, news had reached the local government offices and officials had come to see the work, been impressed, and offered resources for the job.

As with many other similar stories, part of the learning is about how to get something done, and part of it is about the capabilities of the people themselves. The self-esteem generated by making a difference on their own initiative fed back along with the learning from action to boost their confidence to take further initiatives. In that village self-help groups had started, so new sources of funds were accumulating to take further steps in their development.

I concluded the earlier example of Jubilee 2000 with attributes of critical thinking: that it applies systems thinking, it is disruptive and is based on cycles of action and reflection. I want to add, on the basis of the Ethiopian case study, two further related attributes: leadership and facilitation.

Whilst many of the other attributes are often properties of groups, these last two are about individuals. The self-organised learning in Gale Warego relied on the facilitation of staff from the local NGO. They did not tell people what to do, but they enabled them to pursue the process. Within the village leaders emerged who *did* cajole and envision their fellow villagers. These individuals are important, as were Dent, Peters and Carter in initiating the Jubilee 2000 programme which would ultimately mobilise hundreds of thousands of people.

## Critical thinking for everyone

I started out by saying that I did not have *the* answer to the question of how to achieve tipping points and change in seemingly intractable situations. I have shown in one global and one local situation how many different views of the principles of critical thinking converge. They highlight the importance of critical thought based on systems thinking, looking for ways to achieve disruption through cycles of action and learning and through placing a value on the roles of both leaders and facilitators. The title of this talk comes from Donald Schon's book of the same name and he argues strongly that being a reflective practitioner is not optional; it is essential to professional practitioners and all concerned with change – to all of us here. If that is my message then I ought to apply it to GNDR; so here are a few thoughts on how we apply it to our own work.

## Critical thinking about Global Network for Disaster Reduction

Recognising that institutions such as UNISDR and frame­works such as their Hyogo Framework for Action (HFA) on disaster risk reduction are failing to break through the boundaries and achieve substantive change, what can we draw from the principles of critical thinking?

Firstly, that we need to move away from a narrow focus on environmental, large-scale disasters and focus on the whole system. Doing so we have found that actually most losses result from multi-factor small-scale disasters. In pilot work we are currently doing in South America, when we ask people to prioritise risks and impacts, ‘domestic violence’ often appears in the top five. You will not find that in the HFA priorities for action.

Secondly, that we have to understand how to disrupt the system to achieve change. This is not a malicious act, it is a responsibility. The Jubilee 2000 case study illustrates the fact that the system cannot disrupt itself, disruption has to come from outside. As with Jubilee 2000, it is intriguing to note that UNISDR was very supportive of the establishment of GNDR even though it was clear that its role would be to criticise them. As with the INGOs grouping round Jubilee 2000, maybe there was some recognition that a separate entity was needed to create disruptive change.

Thirdly, we have to reflect on our action. Our early reports and campaigns were impactful, leading UNISDR's head to say ‘you have clearly through this work and this product moved the agenda forwards considerably’ (*Margareta Wahlstrom's address to the GNDR Global Workshop* 2010). More recently we have found the impact of our messages blunted as they learn how to deal with us. We need to reflect on our actions, thinking critically to find new entry points.

Finally, we need to raise up and support leaders who can inspire and facilitators who can mobilise – particularly at national and regional level. That is some of our work in progress as we learn to act as reflective practitioners, applying critical thinking in our network.

The problem I posed was illustrated strikingly at global level by the massive investment in development aid failing to drive down the disaster losses curve. At this and all other levels dogged activism and investment of resources are not sufficient to break through the boundaries and achieve transformational change. I have shown through case studies at international and local level how critical thought can lead to breakthroughs, transformation and progress and suggest that, as practitioners in every field, it is part of our role and duty to exercise this skill.

I hope that Pat Reid would recognise and agree with at least some of the ideas presented here and also that you find them useful in your own work as change agents.

## Notes

1.
http://www.globalnetwork-dr.org

2. OECD expenditure tables downloaded from http://www.oecd-ilibrary.org/development/development-aid-total-official-and-private-flows_20743866-table5

3. Estimated damage caused by reported natural disasters 1975–2011. Chart downloaded from www.cred.org

4. Hyogo Framework for action, downloaded from http://www.preventionweb.net/english/professional/publications/v.php?id=1037&pid:22&pif:3

5. Views from the Frontline has conducted questionnaires and consultations with over 100 000 people in disaster affected communities and presents reports of their perspective: http://www.globalnetwork-dr.org

6. Information from personal communications with Isabel Carter and others at Jubilee (2000) and from Clark (2003).
